# An Essay on the Mechanism of Parturition

**Published:** 1829-07-01

**Authors:** 


### AN ESSAY ON THE MECHANISM OF PARTURITION, FROM THE GERMAN OF C. F. NAEGELE, PROFESSOR OF MIDWIFERY AT HEIDELBERG. By *Edward Rigby,* M. D. 12mo. pp. 166. London, 1829.

THE process or mechanism by which Nature, in ordinary or common cases, accomplishes parturition, was for a long period of time involved in great obscurity; and the practitioners of midwifery, who were often right in their mode of managing extraordinary and difficult cases, were continually doing wrong in treating those that were simple and natural. The error committed was usually that of interfering with nature, and endeavouring to hasten delivery; and this interference was dictated by the unnecessary fears of those who were not only ignorant of the true position of the fœtus in utero, but of the direction of the head as it passes through the pelvis, and of the powers by which it is compelled to descend.

It might be supposed that Mauriceau, who possessed an acute and investigating mind, and who had very extensive opportunities of observation, would have discovered the true position of the fœtus in utero, and of the head at the time of birth, but he swerved not from the opinion which had been handed down by tradition, and does not appear to have entertained a single doubt of its accuracy, and thus his authority tended to confirm a belief which, as it was erroneous, could not lead to right practice.

In 1742, an opinion was promulgated by Fielding Ould, of Dublin, which, though it did not teach the exact truth respecting the mode of presentation of the head, yet approached very near to it. In his "Treatise of Midwifery," he informs us that, being at a labour at Paris, which had been expected to be soon over, but in its progress grew very tedious, it was remarked by the attendants, that the head of the child was so disposed, as for its chin to be placed in the direction of its shoulder, and this was supposed to indicate a preternatural position of the head. Ould thought otherwise, and maintained that this was the right direction, to confirm which, he says, "I made the strictest examination of every woman which I either delivered or saw delivered during my continuance at Paris, which perfectly convinced me of the truth of what I suggested."

This suggestion of Ould led other practitioners of midwifery to a more close investigation of this important subject, and it was soon ascertained, that if the position described by Ould was not quite right, it was not much wrong; and from this time, not only did a more simple and excellent method of managing natural labours begin to be adopted, but the practice of midwifery with regard to preternatural and instrumental cases underwent a great improvement. A stronger proof can hardly be required to demonstrate the benefits which are likely to accrue from establishing facts, and deducing practical rules from accurate observation.

Dr. Rigby, the translator of Professor Naegelè's Essay on the Mechanism of Parturition, observes very truly, in his preface, p. ix, that—

"An intimate acquaintance with the mechanism of child-birth is of the highest importance to the accoucheur; it is the foundation on which all his practical knowledge must be grounded; for without it he can have no positive data, from which he may be enabled to draw indications for the course of treatment necessary to be pursued. Uncertain whether any circumstance that may present itself to his observation is to be regarded as unusual, or merely as the result of changes inseparably connected with this natural process, he is unable to draw the important line which is the boundary between health and disease, and to decide in what cases he may confidently leave nature to her own powers, or where and at what period it will be necessary to bring in the aid of art to her assistance.

"An accurate knowledge of the mechanism of child-birth is indispensably necessary in performing the various operations of midwifery, more especially in applying the forceps and in turning; the former, which is one of the simplest and most valuable operations known, becomes in the hands of an ignorant practitioner not less cruel than dangerous; the latter, of itself more difficult and complicated, and infinitely more dangerous to the child's life, depends for its success solely on an intimate knowledge of the relations between the maternal passages and size of the child, as well as on the state in which nature is to assist or retard the completion of this operation.

"An accoucheur who is not well acquainted with the mechanism of parturition, may be fairly said to be in the dark. He can only be compared to the sailor, who, putting to sea without a chart, expects to be blown across the wide ocean to the place of his destination, ignorant of the nature and situation of the shoals and sunken rocks, that may interrupt his course."

And being aware that in his own country, there was no single work which distinctly treats upon this mechanism, he has published in an English dress Naegelè's exact remarks.

Professor Naegelè has had a very extensive practice in midwifery, and the observations with which this little work abounds, shew clearly that he has taken great pains in investigating all the phenomena of natural parturition. He describes the descent of the head through the pelvis as it occurs in four several positions, to which number he limits the varieties differing in this respect from Baudilocque, and the French writers, who describe six.

In detailing the particulars of these four positions of the head, and in enumerating the means by which each is to be detected, the author introduces many useful practical remarks, the result of personal experience, which are well deserving the attention of the young practitioner. In England, especially, accoucheurs are too often inattentive to the mode in which the head of the child comes forward during the early part of labour; and it is by no means improbable that they often embarrass themselves in consequence of this want of care. If the true position of the child's head in the first stage of labour were accurately made out, it would often lead to a more correct prognosis as to the probable duration of the labour. The obstetrician would often be spared the necessity of remaining hour after hour in unnecessary attendance, and would often escape the more unpleasant charge of having absented himself, and neglected his patient, at a time when his services were positively required, and anxiously, but in vain, invoked. A more exact inquiry into the position of the head would likewise prevent those occasional mischievous consequences which result from the use of instruments; for we have reason to believe, that not unfrequently the aid of instruments is employed, or rather attempted, when the head of the child is in no fit position for their use, to the danger of the mother, and the disgrace of the practitioner; and we have had, on other occasions, reason to lament that the judicious use of instruments had not been more timely resorted to, for we have known them delayed so long as to fail altogether in producing any beneficial object.

Professor Naegelè has not confined himself to an explanation of the mechanism of labour in head presentations, he has likewise detailed the process in cases of nates presentations, and we are inclined to think, that his observations on these are more interesting than those which relate to the presentation of the head.

On the whole we consider this little book deserving of being well studied. We must confess that the student will not find in it all the attraction of language which tends to make study inviting. It is rather a *dry* book; the German idiom is too much preserved in the translation; but the student will be amply repaid for the pains he bestows upon it, by the solid information he will gain.

